# Imaging of Transmetallation and Chelation Phenomena Involving Radiological Contrast Agents in Mineral-Rich Fruits

**DOI:** 10.3390/tomography8030114

**Published:** 2022-05-23

**Authors:** Subhendra Sarkar, Zoya Vinokur, Bleidis Buitrago, Lin Mousa, Hayley Sanchez, Analia Basilicata, Jodi-Ann Douglas, Seanetta Reddock

**Affiliations:** Department of Radiologic Technology & Medical Imaging, New York City College of Technology, City University of New York, New York, NY 11201, USA; zvinokur@citytech.cuny.edu (Z.V.); bleidis.buitrago@mail.citytech.cuny.edu (B.B.); lin.mousa@mail.citytech.cuny.edu (L.M.); hayleysa14@gmail.com (H.S.); analia.basilicata@mail.citytech.cuny.edu (A.B.); jdouglas@citytech.cuny.edu (J.-A.D.); shumphreys@citytecch.cuny.edu (S.R.)

**Keywords:** GBCA, transmetallation, environmental toxins, mammographic soft X-ray, gadolinium, iodinated contrast, X-ray, environmental pollution, radiological contrast media, chelation, T_1_ and T_2_ relaxations, contrast induced toxicity, magnetic resonance imaging

## Abstract

Exogenous heavy metals or non-metallic waste products, for example lanthanide or iodinated contrast media for radiological procedures, may interfere with the biochemical pools in patients and in common food sources, creating an excess buildup of exogenous compounds which may reach toxic levels. Although the mechanisms are unknown, our experiments were designed to test if this toxicity can be attributed to “transmetallation” or “chelation” reactions freeing up lanthanides or chelated transition metals in acidic fruits used as phantoms representing the biologically active and mineral-rich carbohydrate matrix. The rapid breakdown of stable contrast agents have been reported at a lower pH. The interaction of such agents with native metals was examined by direct imaging of contrast infused fresh apples and sweet potatoes using low energy X-rays (40–44 kVp) and by magnetic resonance imaging at 1.5 and 3T. The stability of the exogenous agents seemed to depend on endogenous counterions and biometals in these fruits. Proton spin echo MR intensity is sensitive to paramagnetic minerals and low energy X-ray photons are sensitively absorbed by photoelectric effects in all abundant minerals and were compared before and after the infusion of radiologic contrasts. Endogenous iron and manganese are believed to accumulate due to interactions with exogenous iodine and gadolinium in and around the infusion spots. X-ray imaging had lower sensitivity (detection limit approximately 1 part in 10^4^), while MRI sensitivity was two orders of magnitude higher (approximately 1 part in 10^6^), but only for paramagnetic minerals like Mn and Fe in our samples. MRI evidence of such a release of metal ions from the native pool implicates transmetallation and chelation reactions that were triggered by infused contrast agents. Since Fe and Mn play significant roles in the function of metalloenzymes, our results suggest that transmetallation and chelation could be a plausible mechanism for contrast induced toxicity in vivo.

## 1. Introduction

### 1.1. Radiological Contrast Agents and Transmetallation: An Environmental Issue

Radiological contrast agents have significantly increased the diagnostic utility in the past several decades as well as the buildup of harmful medical waste. However, the risk and mitigation steps are not yet understood without more data on how these agents affect enzyme actions and homeostasis. Out of 75 million yearly CT scans in the US, about half are performed with iodinated X-ray contrast agents [1] that frequently cause mild to moderate adverse reactions and life threatening events occurring at a rate of 1/10,000 [2]. Recently, gadolinium based contrast agents (GBCA, with central lanthanide group metal ion) for MRI procedures have also been implicated as serious micro-contaminants to the ecosphere [3,4]. Following the reports of unintended brain and bone deposition of Gadolinium-based contrast agents [5,6], the European Medicines Agency has suspended the marketing authorization for three of the eight available intravenous Gd formulations and limited two more to only liver imaging when justified. The US Food and Drug Administration (FDA) did not recommend any product withdrawals but added new warning labels that advised caution [7].

Gd^3+^ has an ionic radius of 0.99 Å, almost identical to that of divalent Ca^2+^, and is the main reason for the high toxicity in biological systems. Gd^3+^ competes with Ca^2+^ in all enzymes that require Ca^2+^ for proper function, and Gd^3+^ binds with much higher affinity (an example of transmetallation) and alters the biological process catalyzed by that enzyme [8].

Transmetallation is a type of organometalic reaction or mechanism step in which a metal bonded to original ligands is exchanged for ligands in another metal. It is believed that transmetallation happens for lanthanide contrast agents in vitro [9,10] as well as in vivo [11]. Gd concentrations in surface waters (up to 1 mg/L), in sediments (up to 90 μg/g), and in living organisms have been observed [12]. Such studies typically use destructive analytical tools and do not map spatial distribution of transmetallation regions. Our work is based on non-invasive imaging in real time demonstrating chelation or transmetallation with active biometals in bulk carbohydrates of apple and sweet potatoes. 

### 1.2. X-ray Absorption by Endogenous and Exogenous Minerals in Carbohydrate-Rich Fruit Matrix 

X-ray interactions with atomic electrons of a sample can be classified in three groups: Photoelectric absorption (PE, probability~Z^4^ at the clinical energy range, <200 keV); coherent or elastic scattering (Raleigh type, probability~Z^2^) and Inelastic scattering (Compton type, probability~Z, the proportionality refers to atomic cross sections that are approximately constant for all elements. The linear attenuation coefficient depends on the photon energy of the X-ray, E_eff_ or mean energy in clinical poly energetic X-ray beams (approximately 1/3 of peak energy, kVp), the electron density of the material, ρ_e_ and the atomic number of the material, Z and can be expressed as the sum of negligible Compton and dominant PE contributions as follows [13,14]:

μ = ρ_e_[a + bZ^3.8^/E_eff_^3.2^] (1)

where a is associated with inelastic Compton scattering that is not sensitive to the type of absorbing atom while b is a constant related to PE absorption and is the main source of contrast at low keV. Such PE step-functions cause increased absorption at specific X-ray energies (approximately two-fold for low Z atoms, minerals in our samples) and five-fold for high Z (Gd and Iodine agents). Composite fruit matrices with endogenous and exogenous mineral distributions may be described with a composite mass attenuation coefficient (μ) equal to a mass weighted sum from key composite elements. However, the most abundant endogenous elements in food and in the human body are O, C, H, N, Ca, P, K, S, Na, and Cl, none of which have sharp K-edges close to the mean energy of 10–15 keV in our experiments except transition metal atoms (Table 1 and Table 2), and may not be detectable by clinical X-ray systems [15].

### 1.3. Positive and Negative Signal Enhancement in Proton MRI by Endogenous and Exogenous Minerals

Proton MRI offers different kinds of information and more specificities than X-ray or CT in the sense of richer gray scale for inherent soft tissue image contrast and its discrimination power based on spin-lattice relaxation properties, vascularity between regions and concentration of infused contrast material i.e., between areas that may have higher degree of perfusion of external contrast and the ones with lower perfusion. However, MR image regions are also prone to signal loss due to tissue inhomogeneities and excessive concentration of paramagnetic materials. In the original concentration range (0.25–1.0 M) gadolinium agents are highly paramagnetic and when a small amount is administered intravenously, the MR signal within the vessels and in perfused tissues show increased signal due to positive contrast enhancement from the automatic dilution event of 100–200-fold within the left ventricle of the patient (thus reduced paramagnetism). Ex vivo gadolinium application at higher concentration however can cause signal loss adjacent to local paramagnetic substances and subsequent proton T_2_ and T_2_* reduction from excess gadolinium accumulation as we believe to be the case in Figure 3. Paramagnetics induced signal change by varying exogenous contrast concentrations may help distinguish highly paramagnetic and weakly paramagnetic endogenous species when the former causes signal loss (negative enhancement) and the later causes signal gain (positive enhancement). In our experiments, either an identical amount of Gadavist or no Gadavist was added in various fruits with higher or lower amounts of endogenous Fe and Mn ions (Figure 3).

### 1.4. pH of Host Matrix and Influence on the Stability of Exogenous Contrast Agents

Schmitt-Willich [16] proposed that the stability of linear contrast agents that undergo fast breakdown can be assessed at high (basic) and physiological pH (neutral) using a fast kinetics model and extrapolated it to macrocyclics that require a slow kinetics approach due to their ultra-stable nature at physiological pH. On the other hand, starting at a pH of 1, where the macrocyclics release Gd quite fast, Schmitt-Willich [16] used a fast kinetics approach and extrapolated the half-lives of Gadolinium at a physiological pH of 7 to be >1000 yrs.

We believe that such an extrapolation from pH 1 to 7 is unreliable without verifiable low/mid-range results at pH 3–5. Toward that goal, several fruit phantoms can be used that are mildly acidic and may serve as excellent in vitro models at this low/mid pH range. At low pH there seems to be proton assisted reaction possible that destabilizes Gadolinium complexes to a half-life of just a few hours instead of years [11]. The presence of various counterions is important; for example, hydronium ions in acidic fruits may weaken the Gd-ligand bonds and allow the release of free Gd.

### 1.5. Residual Radiologic Contrast In Vivo and Detection Limits

Lusic et al. [17] have computed an effective concentration limit for iodinated contrast agents for detectability by X-ray based CT in molar range while for MRI, the detection limit seems to be in the μmolar range. Regarding absolute sensitivity, the detection limit for the MRI contrast agent Gd-HPDO_3_A in mouse skeletal muscle was reported to be 30 µM [18]. Detecting the paramagnetic concentration at micromolecular range by MRI will require a new set of experiments that provide estimates for realistic situations. Using dedicated MR hardware and a more than clinically feasible time frame, animal imagers may detect perhaps tens of micromolar concentrations of residual Gd.

Inherent tissue contrasts are quite different in MR and CT images [1,17] and have not been well understood. Note that transmetallation in brain as well as in species along the seabed have been demonstrated by invasive analytical tools, while no prior studies have been done to establish feasibility or demonstrate spatial distribution of transmetallation byproducts.

Researchers found that at body pH a small fraction of the injected Gadolinium complexes break down and release free Gadolinium, mainly for linear agents but also for macrocyclics. Using mass spectrometry and transmission electron microscopy (TEM) in rat models injected at the human clinical equivalent dose of 0.1 mmol/kg, McDonald et al. [19] quantified residual Gd at several μg per kilogram of brain tissue while for rest of the body with higher accumulations. Using scanning electron microscopy (SEM) and energy dispersive X-rays (EDX) Thakral et al. [20] identified insoluble Gd-containing deposits on skin samples from 29 patients with a history of NSF from macrocyclic GBCA administrations. Such free Gd deposits confirm the removal of Gd from infused macrocyclic structures by other metal ions (indicative of transmetallation). Using chromatography in sacrificed mouse brain, Kartamihardja et al. [21] detected high Gd concentration of the order of μmoles in the meninges and in olfactory bulbs demonstrating intact blood brain barrier penetration even from presumably stable macrocyclic agents.

### 1.6. Focus of This Work

A motivating factor for our work is that for both X-ray/CT and MR contrast families the mechanism of tissue distribution or contrast breakdowns is not understood. Hence we have focused on the interaction of both types of contrasts with native biometals in two carbohydrate models at mid-to-low pH ranges. Detection of less abundant elements (like endogenous transition metals in our model fruits) is difficult. That was the first step we wanted to explore, noting that k-edge advantages for transition metals exist over alkali and alkaline earth at the lower end of clinical X-ray energies where photoelectric effects dominate. It was also noted that whole fruits required higher kVp X-ray beams to penetrate and did not show mineral distribution while lower end of kVp (at 40 kVp for planer X-ray systems and 20–30 kVp for mammographic X-ray units were able to differentiate between bulk matrix and mineral centers in our fruit models.

Endogenous biometals in various fruits, for example, iron, calcium, manganese, magnesium and copper, have high affinity for the exogenous contrast ligands while Lanthanides (Gadolinium in our MR contrast complexes) have even higher affinity for the synthetic ligands in the Gd complexes. However, counterions, as hydronium ions in low pH matrix, can weaken the ligands, while a large number of divalent or trivalent transition metal ions may shift the lanthanide-ligand equilibrium for gadolinium, allowing transmetallation to be favored. Similarly, iodinated X-ray contrast media (bond energy >50 kcal) [22] may be able to attract highly charged cations from the fruit metalloenzymes by chelation and essentially destroy enzyme structures and simulate contrast toxicity in vivo.

In a prior work we explored for the same agent using X-ray imaging, the time course of iodinated species diffusion at the low pH environment of pineapple matrix, and observed both fast and slow diffusing X-ray absorbing species, fast attributed to lower molecular weight fragments [23]. The current work presents the first imaging observation of transmetallation involving gadolinium and chelation involving iodinated contrasts. No attempt was made to identify the oxidation state or concentration of such transmetallation products using chromatography or electron microscopy since such analytical tools do not preserve endogenous biochemical states.

## 2. Materials & Methods

Experiment design and sample preparation.

### 2.1. Low Energy X-ray Experiments

**Figure 1:**
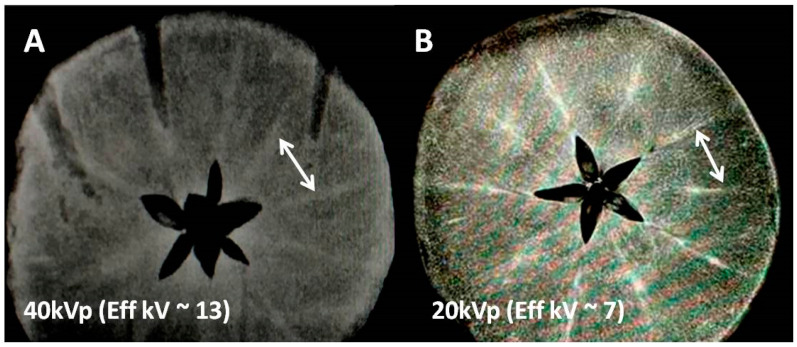
X-ray images of the mineral-rich apple at two different X-ray energies showing mineral conspicuity as a function of mean X-ray energy. (**A**) Non-contrast X-ray image at 40 kVp/10 mAs (with mean energy approx. 13 keV) using a clinical scanner; two typical high attenuation tracks marked by a white arrow indicate either K or Fe concentrated regions (Table 1). (**B**) Another apple slice showing the same mineral tracks at higher mineral conspicuity presumably due to lower kVp (soft) mammographic X-ray beam (20 kVp/10 mAs with mean energy approx 7 keV, close to k-edges of transition metal ions but far from k-edge of K ions, Table 1).

**Figure 2:**
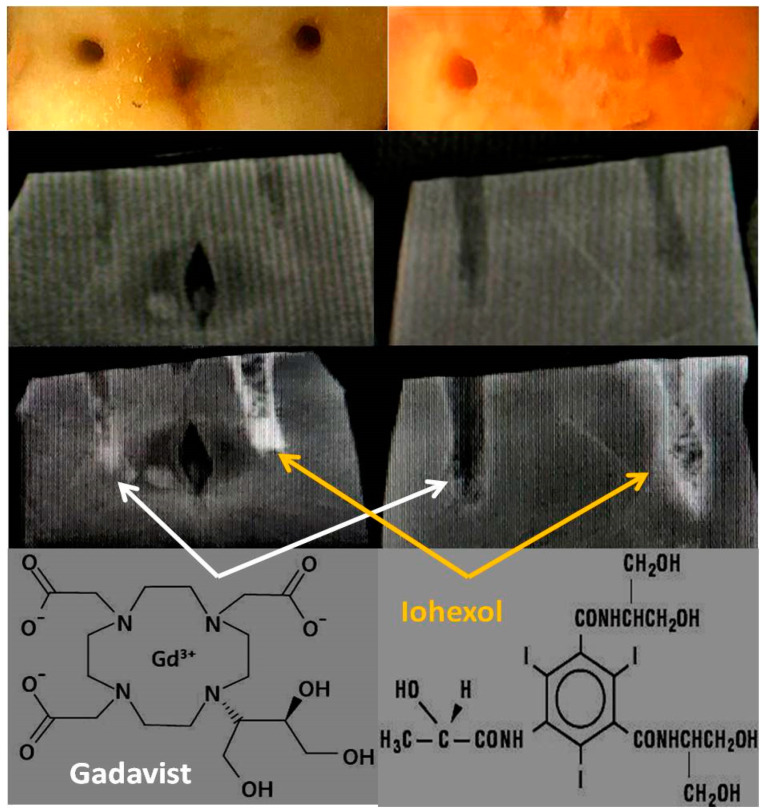
X-ray images of the mineral-rich model fruits and vegetables. 1st row: Cut samples: apple (left) and sweet potato (right) and manually carved holes (2 × 2 × 10 mm^3^ or 0.04 mL for apple and approx 3.3 × 3.3 × 20 mm^3^ or 0.2 mL for sweet potato). 2nd row: Pre-contrast X-ray images at 44 keV/15 mAs by a clinical scanner. 3rd row: Post-contrast X-ray images showing Gadavist is perfused in the left wells (white arrows) and Iohexol in the right wells (yellow arrows) for both fruits. 4th row: Molecular structures of both of the contrast media.

For two tissue regions (consisting of two different elements) low energy X-ray absorption in each tissue is dominated by photoelectric effects and depends on the fourth power of the tissue atomic number (Z^4^) i.e., X-ray absorption is greater for the heavy atoms. This factor theoretically can create an inherent tissue contrast spanning several orders of magnitude (6^4^ using in vivo tissue effective Z of 6 and 53^4^ for iodine with Z = 53). However, creating differential X-ray absorption within the same tissue for light weight minerals (e.g., iron, calcium, magnesium, potassium etc.) can be substantially difficult due to the volume averaging of minute amounts of such metal ions in normal tissue. In our experiment design we kept the X-ray peak energy low, 40/20 kVp (Figure 1) 44 kVp (Figure 2), respectively. This allowed the mean energy range to be just above the K and L edges of transition metals but still far above the most abundant alkaline metals. Hence, as verified by MR images we are able to favor detection of the Fe and Mn rich tracks by MRI (Figure 1), while arguably discriminating alkali and alkaline earth elements with mean X-ray energies (15 and 7 keV) far above the alkali elemental k edges (approximately 2–3 keV) in Figure 1 and Figure 2. We explored the detectability limits of clinical X-ray systems for endogenous biometals that are lightweight and less abundant and hence hard to detect for the above reasons.

### 2.2. MRI Based Detection Experiments

This work was designed to conduct contrast enhanced MRI experiments at the natural state of various fruits to test the potential interaction by endogenous biometals with externally infused Gadolinium macrocyclics or iodinated CT contrasts. The plausible outcomes were:

1. endogenous minerals from the sample fruits may free up toxic metal ions like Gadolinium that may precipitate as Gadolinium salts and have been observed in certain in-vivo applications, or
2. fruit biometals may get chelated by the iodinated contrast agent or by macrocyclic Gadolinium ligands and may be detectable by X-ray or by MRI based on spin-lattice or transverse relaxations.
3. none of the consequences above take place at the concentration level detectable by imaging.

Several researchers have observed T_1_ and T_2_ shortening in vivo for proton MR images in the presence of iodinated contrasts [24,25,26]. While they suggested that iodine may be restructuring the tissue water, we feel these prior works [24,25,26] have observed chelation phenomena by iodinated complexes that pulled native paramagnetic minerals to the iodine sites leading to T_1_ and T_2_ shortening of surrounding bulk, and seem to directly support the chelation demonstration in our experiments that is, MR detection of paramagnetics accumulation when iodinated contrast was the sole exogenous agent.

The MRI experiments were designed based on the magnetic susceptibility of iron oxidation states. Fe^2+^ complexes have low magnetic susceptibility (often diamagnetic due to paired electrons or weakly paramagnetic, depending on ligands) and cannot substantially affect nearby tissue proton MRI signal. On the other hand, Fe^3+^ complexes possess several orders of magnitude higher magnetic susceptibility (paramagnetic due to lone electron) and hence influence nearby tissue contrast quite strongly with enhanced T_1_ relaxation (positive contrast, if diluted) or by enhanced T_2_ loss (negative contrast enhancement) and we believe that this has been accidentally observed by prior workers [24,25,26] when iodinated agents existed in tissues of MR interest like stroke brain.

To identify the role of endogenous paramagnetic moieties in the absence of Gadolinium addition (that is to stage only chelation by iodinated agents and not transmetallation with Gd complexes), we have specifically performed the Iodine only experiments by MR, without infusing any Gadolinium agents. We believe a small amount of paramagnetic substance accumulated since the iodinated contrasts in general are chelating agents (Fe and/or Mn). Next, note that apple does not have Mn but only has Fe in addition to other non-magnetic minerals while sweet potato has both Fe and Mn. We also believe that the T_1_ and T_2_ shortening observed in vivo with iodinated contrasts as noted in prior works is not simply due to the restructuring of water of hydration but most likely due to the simple chelation accumulation of a small amount of paramagnetics at the contrast addition sites.

We explored the feasibility of image-based detection of chelation and transmetallation phenomena triggered by radiological contrast agents. Since linear contrast agents break down faster than macrocyclics and have been almost delisted from the markets, our experiments focused only on macrocyclic MR agents that are known to be safer than linear agents, but the degree of safety and the factors leading to unsafe breakdown have not been understood.

### 2.3. Exogenous Contrast Agents (Environmental Stressors) and Imaging Instrumentation

#### 2.3.1. X-ray Equipment

The imaging technique was optimized to be 44 kVp and 15 mAs (focal spot size, 0.25 mm) at 40” SID using a digital X-ray system from Quantum Medical, Ronkonkoma, NY, USA.

#### 2.3.2. MR Hardware

A 1.5 Tesla Solara MR (Siemens Medical Solutions, Erlangen, Germany) system equipped with a 20 channel phased array head coil for receiving and a 16 channel CP body coil were used for radiofrequency excitation and refocusing in transmit mode.

#### 2.3.3. Radiologic Contrast Media (as Environmental Stressors)

Gadavist (Bayer Healthcare, New York, NY, USA) C_18_H_31_GdN_4_O_9_ or Gadobutrol, a widely used paramagnetic complex consisting of gadolinium (Gd^3+^), was chelated with the macrocyclic compound dihydroxy-hydroxymethylpropyl-tetraazacyclododecane-triacetic acid (butrol) at a concentration of 1 mmol/mL or approx 157 mg of Gd/mL and for Iohexol or Omnipaque 300 and 350, C_19_H_26_I_3_N_3_O_9_ contained 300 or 350 mg iodine/mL of distilled water.

#### 2.3.4. MR Sequences

A T_1_-weighted turbo spin echo sequence was used for imaging model samples in this study with the following parameters:

TR/TE/Flip Angle/Turbo factor/FOV/Matrix/Voxel Resolution/Bandwidth: 300 ms/11 ms/70 degree/3/128 × 128 mm^2^/256 × 256/0.5 × 0.5 × 4 mm^3^/200 Hz/pixel.

And a T_2_ weighted spin echo sequence with the following parameters:

TR/TE/Flip Angle/Turbo factor/FOV/Matrix/Voxel Resolution/Bandwidth: 2000 ms/70 ms/50 degree/1/128 × 128 mm^2^/256 × 256/0.5 × 0.5 × 4 mm^3^/200 Hz/pixel.

The samples were positioned at the isocenter of the magnet bore inside the head array RF coil and were shimmed to produce a spectral line width of 80–90 Hz at 1.5T and 120–130 Hz at 3.0T to increase detection sensitivity.

#### 2.3.5. Sample Preparation

Conventionally grown red delicious apples and Beauregard, USA variety sweet potatoes were used as model mineral-rich fruits [27,28,29] (Table 1 and Table 2). Freshly cut 1 cm thick fruit samples with 2–3 mm (dia) × 10–20 mm (deep) carved wells (volume 2 × 2 × 10 mm^3^ or 0.04 mL in apple and 3 × 3 × 20 mm^3^ or 0.2 mL for sweet potato) were used for infusing several radiological contrast media as shown in Figure 1. The contrast materials for infusion were Gadavist (Bayer Healthcare, LLC, New York, NY, USA) and Iohexol (Omnipaque 300 and 350, GE Healthcare Ireland) that were filled in the pre-carved wells and time series images were obtained in both X-ray and MR imaging modalities.

## 3. Results

### 3.1. Exogenous Contrast Agents Added to Each Well (Normalized to 0.04 mL Volume)

Gadavist: 0.1 mmol/kg C_18_H_31_GdN_4_O_9_ (MW 605 g/mole) supplied 157 mg of Gd atoms/mL i.e., 6 mg of Gd in 0.04 mL volumes in both the wells of apple and sweet potato.

Iohexol: Omnipaque 300, GE Healthcare Ireland, C_19_H_26_I_3_N_3_O_9_ containing 300 mg iodine/mL supplied 12 mg of Iodine in 0.04 mL volumes in apple and sweet potato.

### 3.2. X-ray Absorption cross Sections for Endogenous Biometals

Considering the Z^4^ dependence in clinical X-ray energy range when X-ray energies are available for photoelectric effects, the absorption cross sections (barns/atom) ∞ Abundance × (Z^4^) and were estimated by adding K, L and M shell photoelectric cross sections [30] for the upper limits assuming photon flux is available slightly above the respective K-edges and are presented in Table 1 and Table 2. These entries are two to seven times less in apple compared to sweet potato for alkali/alkaline earth biometals and one order of magnitude less for transition metal ions. Since the mean energies present in the 40 kVp X-ray beam are in the 10–15 keV range (1/3 of the kVp), far higher than the K-edges for alkali/alkaline earth but not too far off from the K-edges of transition metal ions in selected fresh fruits. Also, if the minerals are concentrated in certain regions, as the X ray and MR images suggest, the local absorption should be much higher than average or global absorption estimates.

The computed entries (barns/atom) are upper limits for cumulative absorption cross sections for various elements assuming that in the poly energetic beams there are abundant photon flux available at energies close to K,L,M -edges for K, L, M shells all of which when added provides a possible upper limit using the full spectrum of energies available all the way up to 40 keV. This is unrealistic but provides a fair comparison between, for example, potassium and iron.

The computed entries (barns/atom) are upper limits for cumulative absorption cross sections for various elements assuming that in the poly energetic beams there are abundant photon flux available at energies close to K,L,M -edges for K, L, M shells, all of which when added provide a possible upper limit using the full spectrum of energies available all the way up to 40 keV. This is unrealistic but provides a fair comparison between, for example, potassium and iron.

### 3.3. X-ray Imaging

Iohexol in the right wells in apple and sweet potato produces localized accumulation of radio-opaque moieties with time (Figure 2). Due to broad band X-ray sources, such X-ray attenuations in the sample from all absorbing materials are detected as one signal and cannot be separated into individual mineral contributions without using filters. In apple and sweet potato, the attenuations are narrowed to two biometals apart from iodine; it is from Ca and K in sweet potato while only from K in apple. Note that the X-ray attenuation contributions from Na, Mg, Mn, Cu and Fe are negligible in both apple and sweet potato due to either low abundance or low K-edges compared to the effective beam energy (approximately one third of the kVp of 44, i.e., 15 keV).

### 3.4. MR Imaging

**Figure 3:**
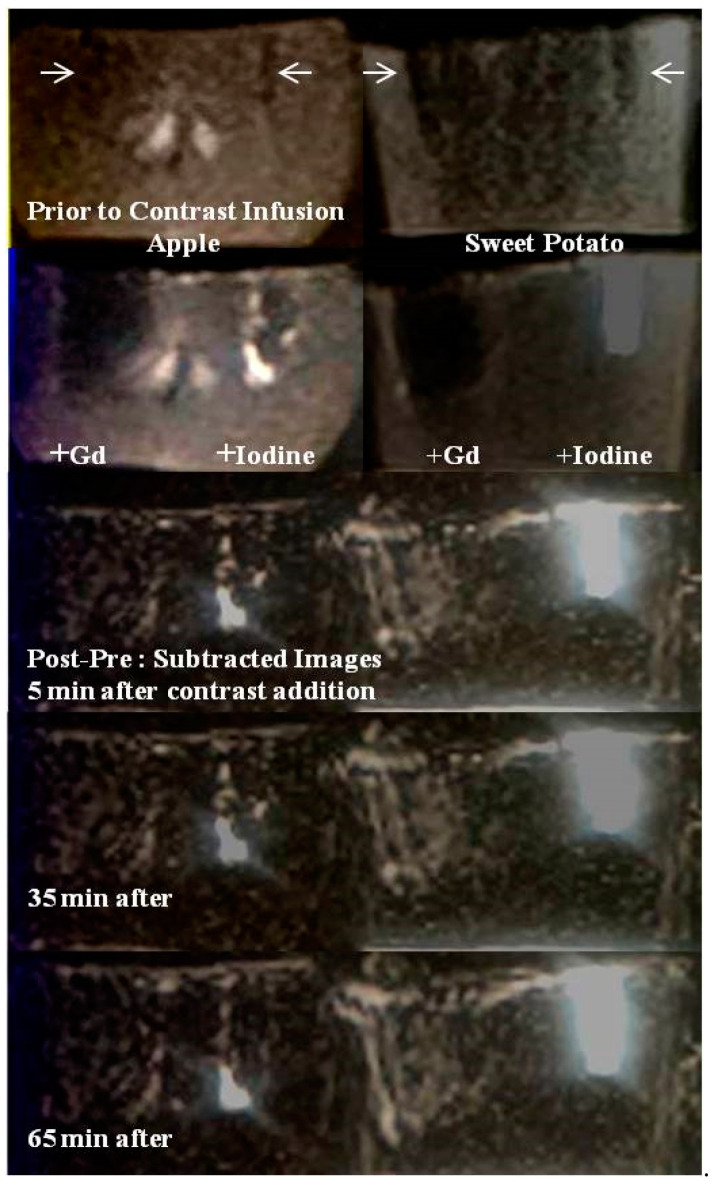
MR images of the mineral-rich model fruits and vegetables. First row: Pre-contrast MR image at 1.5T Siemens system, apple (left) and sweet potato (right). Manually carved wells (2 × 2 × 10 mm^3^ or 0.04 mL volume) are shown (white arrows). Second row: Post-contrast MR image when Gadavist is added at the left wells and Iohexol in the right wells for both fruits. Notice signal loss due to paramagnetic nature of Gd^3+^ and enhanced signal due to Iohexol. Third to fifth rows: Time series subtraction images (post-pre contrast) at time = 5 min, 35 min and 65 min are shown. More pronounced enhancement is seen with Iohexol in sweet potato.

In the left panel of Figure 3, the T_1_-weighted images (top two rows, prior to and immediately after Gadavist and Iohexol addition) show large signal voids in the left, within and adjacent to the Gadavist added wells while on the right, the Iohexol added wells and surrounding bulk matrix show hyper intense signals in both samples. This is better visualized in the (post-pre) contrast subtraction images (bottom three rows, Figure 3).

The signal voids are due to T_2_^*^ shortening of bulk protons by strongly paramagnetic Gd^3+^ in the infused Gadavist. The continued signal enhancement in the carbohydrate protons surrounding the right wells with time are presumably due to a chelation process by Iohexol complexes for extracting paramagnetic metals from the biochemical pathways within both samples. Potential chelated paramagnetic candidates that can induce T_1_ shortening of bulk protons are Fe^2+^ and Mn^2+^ in both systems

The iodine added MRI image was acquired five days post iodinated contrast addition, while contrast was allowed to be absorbed in the walls of the taped wells (generally took a few hours for absorption). Five days post images shows the stable, residual distribution of minerals released by the bulk matrix in response to iodine induced chelation, with no MRI contrast involved. With T_1_ and T_2_ both being bright in these images, one may suspect the accumulation of weakly paramagnetic species in the walls (originating from endogenous pool) that have caused T_1_ shortening and T_2_ lengthening of bulk protons. These are suspected to be low concentrations of Fe and/or Mn species chelated by iodinated media.

Top row: T_1_-weighted image (parameters in the text) showing rim enhancement of residual iodinated species presumably containing a paramagnetic substance that lowered the T_1_ relaxation times for both wells of both fruits. This also shows multiple focal and diffuse regions of such paramagnetic species distribution at the interior of the fruits that should be Fe ions which were probably removed from biochemical reservoirs and that are present in Fe^2+^ or Fe^3+^ forms.

Middle row: T_2_-weighted image (parameters in the Methods section) showing high signal from long T_2_ species near the wells and almost total signal loss from short T_2_ species at the interior for both of the fruits. Such paramagnetic effects seem to suggest iron chelation by iodinated agents.

Bottom row: After MRI, the fruits were cut open, and the bottom row shows an optical photograph of a residual iodine stain in the surrounding matrix of the wells. The wells were fully dry with no fluid trapped or any sign of degradation since iodine kills bacteria. Omnipaque 350 shows darker stains, thick arrow (350 mg/mL of iodine present as opposed to 240 mg/mL in Omnipaque 240, thin arrow).

### 3.5. Transmetallation Detection Limits

Although establishing the lowest detection limits was not a part of this work, the following conservative sensitivity limits can be reported:

For the alkaline earth minerals (Ca, K), mid-clinical keV range X-ray detection was possible around Gd wells where the only detectable elements were Ca and K at concentrations of tens of mg/100 g bulk sample or one part per 10^4^. However, the mammographic X-ray energy range is able to increase the metal ion sensitivity to some extent compared to non-mammographic clinical systems. For a paramagnetic mineral (Fe, Mn), the detection was possible near iodinated wells where the only minerals available were transition metal ions at concentrations of 0.1 mg to 0.01 mg per 100 g bulk matrix or one parts per million using mid-field or 1.5T MRI, and are expected to improve at high fields of 3T and beyond (Table 1 and Table 2).

## 4. Discussion

This work demonstrated that low energy X-rays in mineral-rich fruits can be used to map low atomic weight metals (first row transition elements) that are biometals in food and animal tissues and are important for functions of metalloenzymes. In particular, we believe some of the transition metals were extracted out of their biochemical pools due to transmetallation and chelation triggered by radiological contrast media. Such a mineral redistribution may not be directly observable by high resolution microscopy (SEM, for example) or analytical tools (GC, MS for example) as those are destructive tools and do not retain native biochemical conditions. Solvated proton (hydronium ion, H_3_O^+^, in acidic matrix) assisted breakdown of MR contrast agents at low pH is common and Idee [11] et al. have reported half-lives of several hours at pH 1.0 for stable macrocyclic agents; that is, even macrocyclics are not stable at low pH.

Transmetallation is a relatively new finding that has not been explored in our food sources by imaging tools. The detection ranges for both of these modalities are dependent on paramagnetic states of the metal ions for MR and the k-edges (atomic numbers) of the metal ions for X-rays. Although an MRI can be sensitive, the quantification by MRI is difficult depending on a variety of susceptibility factors including the sequence parameters and field homogeneity. On the other hand, X-ray quantification is direct. Although the present work is not to establish an absolute sensitivity, it demonstrates for the metals in the model matrix (Table 1) detection by imaging as follows: for X-ray, the detection limit for Ca and K is approximately 10–100 mg for Ca and K (due to low detectability by X-rays) while it is 0.01–0.1 mg for Mn and Fe/100 g of bulk carbohydrates (due to high sensitivity for MRI). This translates to a comparative sensitivity of 10^−3^–10^−4^ for medium energy clinical X-rays and a hundred-fold higher sensitivity, approximately 10^−6^ for mid-field (1.5T) MR for transmetallation in carbohydrates.

Nanoparticles uptake, processing and relocation by plants can be studied by optical and electron microscopy to provide information about biometal distribution as reviewed recently by Jitao et al. [31]. Here we demonstrate that using radiological contrast media transmetallation in fresh food sources can be detected by direct mapping of X-ray intensity distribution or by enhanced spin relaxation due to extracted biometals by Gadolinium or iodinate contrast media. Our imaging results seem to confirm a key work on transmetallation when added Gd-DTPA increased the solubility of iron by formation of Fe-DTPA [10]. Although in vivo toxicity of radiological contrast agents is important, focusing on their stability in vivo at physiological pH alone or only at the extreme pH ranges [14], may not explain the roles of endogenous metals on the contrast breakdown rates and mechanisms due to other chelators present [32].

Note that there exist other imaging modalities like the nuclear isotope imaging that are sensitive at μmolar range, and optical imaging tools like fluorescence microscopy are effective even at nanomolar range for the absorbing material. However, the external contrast isotopes infused for nuclear imaging tools do not interact with biometals and hence do not help detect disrupted homeostasis or transmetallation.

The kinetics and chemistry of contrast agents are an active area of development as summarized in two excellent reviews [33,34]. Similar to iron homeostasis, manganese controlled enzyme reactions are also affected by lanthanides and iodine based contrasts currently on market that may be avoided with nanoparticle- based new generations of contrasts [35]. During these development initiatives it is prudent to note that developments in vitro often are not directly applicable in vivo where counterions and physiology are major variables, as stressed in the transmetallation and chelation results presented here.

## 5. Study Limitations and Future Directions

**Figure 4:**
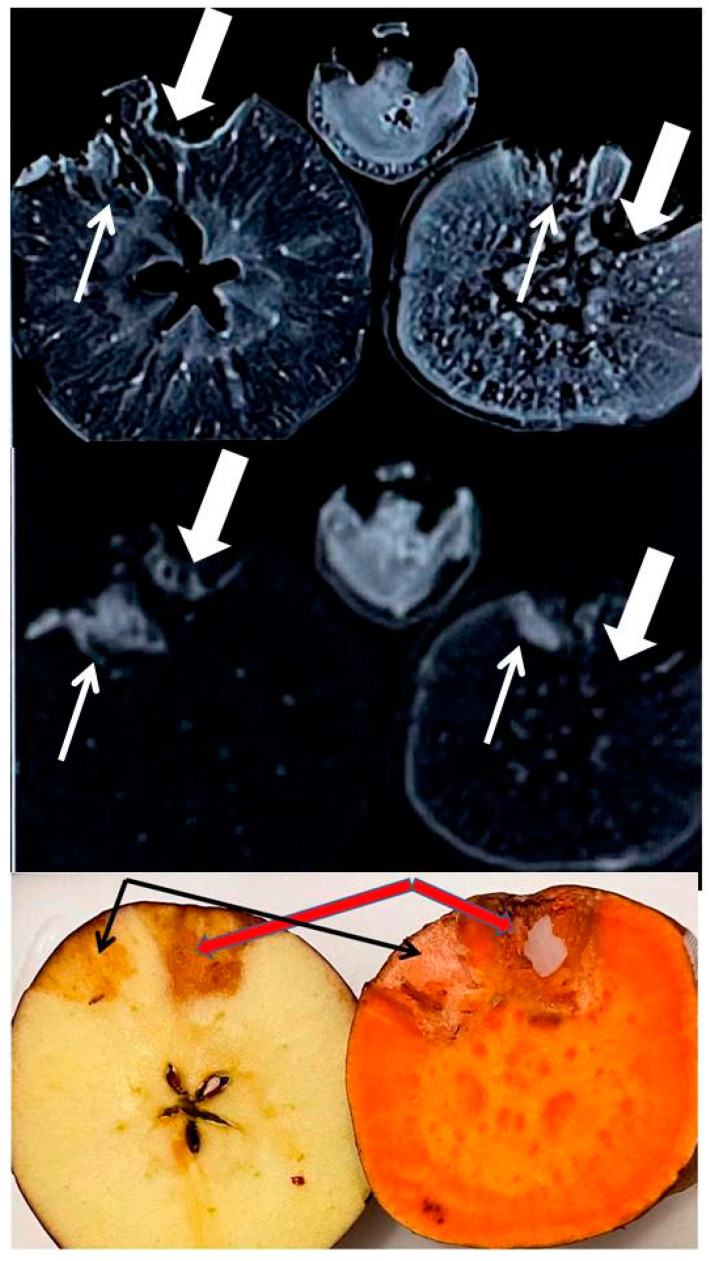
High Field (3T) MR images of the mineral-rich apple (left) and sweet potato (right), each with two carved wells infused five days prior with iodinated contrasts Omnipaque (240, thin arrow and 350, thick arrow). Also shown is the image of a banana at the superior/middle aspect.

The current work barely touches upon the detection limits and does not set the goal for residual Gd detection for the purpose of neuro or nephro toxicity. Our work uses paramagnetic sensitivity advantages to detect only Mn and Fe that has significant implications in enzyme and carbohydrate homeostasis. It seems that the endogenous imaging detection of most of the minerals in native fruits is substantially difficult for clinical MR scanners that are basically vastly abundant proton imagers, while microscopic amounts of minerals are often homogenously distributed at ppm concentrations. Apple seems to be a small exception and has been used to demonstrate the advantages of chelation and transmetallation. It has mineral tracks probably at higher concentrations than the average reported in Table 1. Iodinated and gadolinium complexes are capable of causing the accumulation of paramagnetic or lanthanide elements extracted from the bio environments in both fruits and offer a detectable MRI signal (see both wells in Figure 4 and second wells in Figure 2 for both apple and sweet potato: with only iodinated exogenous input). Since iodine is not MR active, we believe this is due to the accumulation of native Fe or Mn at the iodine sites. This may mean that chelation has created a higher concentration of Fe or Mn and now MR is sensitively detecting those. This is also favoring track detection in apples in Figure 1 where low kVp (20 kVp, mean energy approximately 7 keV) in the mammographic system provides a more favorable K-edge detection for Fe in apple as compared to 40 kVp (mean energy approximately 13 keV).

In these kinds of experiments, the sample preparation needs to be as non-invasive as possible to preserve the biochemical mechanisms and native mineral transport. Also, consistency and reproducibility are very important. Our samples were obtained from two local vendors and one national chain. However, a more representative national or global sample collection is necessary and will be pursued in the next phase. Even performing experiments at lower kVp X-ray systems (mammographic kVp range and below) and stronger magnets (7 and 9T) than the 1.5 and 3T used here will be helpful to extend the detectability limits of light minerals. High resolution CT with 3D reformations should also be considered. Although for clinical CT scanners the lowest kVp available is usually 70–80 kVp, which is way higher than the k-edges of most of the elements of interest, the fully non-invasive imaging could be valuable for the detection of biometals at a native state similar to whole fruit MR. As more contrast agents are offered in the market, we feel the safety and efficacy testing should be extended to different pH levels and include the presence of several counterions.

One may note that the image quality is not high, which is a drawback of this study. However, partial volume effects as well as time averaging may decrease the conspicuity and detectability of time sensitive transmetallation events. Hence, we have imaged at a high speed using 2D slices at 1.5T that cost SNR. 3D sequences that offer higher SNR require imaging of the whole fruit and take a longer time. Our goal was to image fast to follow transmetallation and chelation kinetics and in future compare 1.5T with 3T where image quality will be higher with paramagnetic effects stronger, allowing superior sensitivity.

## 6. Conclusions

While intravenous contrast deposition in human tissue has received significant attention due to nephro-toxicity and accumulation in vivo, the mechanism underlying contrast instability remains unclear. However, counterions and low pH have been known to affect contrast stability, and we concur with that here. We have demonstrated that in a mineral-rich biological matrix at low pH, transmetallation and chelation reactions triggering exogenous contrast breakdowns are a distinct possibility. Our MR results seem to offer greater sensitivity and specificity than X-rays, but the detection is limited only to endogenous Fe and Mn based on their paramagnetism. Fe and Mn play significant roles in the function of metalloenzymes, and our results on model biological matrices although not directly applicable to mammalian tissue, raise transmetallation and chelation involving Fe^3+^ and Mn^2+^ as a plausible mechanism for accumulation and toxicity of medical contrast media in vivo.

## Figures and Tables

**Table 1:** Endogenous mineral content in red delicious apple and estimated amount available for transmetallation or chelation in a thin imaginary cylinder around the wells.

	Minerals in Common Oxidation States	Gd3+ 157 mg/mLV_well_ = 0.04 mL	Iodine 300 mg/mLV_well_ = 0.04 mL	Na+	K+ ^Ұ^	Mg2+	Ca2+	Mn(2+,3+,4+) ^¶^	Cu2+	Fe(2+,3+) ^¶^
Exogenous or Endogenous Minerals										
Content in **mg/100 g or 220 mL** volume of apple (density 0.45 g/mL)		0	0	1	104	5	6	0.03	0.03	0.1
Endogenous content in **μg/mL** of bulk volume in apple		0	0	5	500 ^Ұ^	25	30	0.15 ^¶^	0.15	0.5 ^¶^
Exogenous content in **μg** added to carved wells *(per 0.04 mL of well volume)*		6000	12,000	0	0	0	0	0	0	0
Endogenous content in **μg** in a thin imaginary cylinder (v = 0.04 mL) around the wells		0	0	0.2	20 ^Ұ^	1	1.2	0.006 ^¶^	0.006	0.02 ^¶^
K-edges (keV)		50	33	1	3.3	1.3	3.7	5.9	8.0	6.4
Cumulative X-ray absorption cross sections (barns/atom) assuming X-ray energies available for photoelectric effects σ < = Abundance × (Z^4^) ^§^		-	-	**σ(expt)** **<0.7 ×** **10^6^**	**<<** **65 × 10^6^**	**<<** **0.5 ×** **10^6^**	**<<** **5 ×** **10^6^**	**σ(expt)** <0.05 × 10^6^ = σ (computed)	< 0.1 × 10^6^	< 0.2 × 10^6^

^Ұ^ indicates plausible X-ray absorbers (high abundance or high Z). ^¶^ indicates paramagnetic minerals that alter T_1_ and T_2_* MRI relaxation times of bulk protons. ^§^ denotes inelastic scattering cross sections σ, for absorption at clinical keV range where PE interaction dominates. Note that σ(expt) is much less than the σ(computed) depending on how far off the K-edge a particular metal ion from mean energy of 10–15 keV for beams of 40–44 kVp is. << stresses that the k-edge is far lower than mean energy of X-ray beam; hence σ(expt) << σ(computed) for alkali and alkaline earth; and < stresses that the k-edge is slightly lower than mean energy of X-ray beam; hence σ(expt) < σ(computed) for transition metals.

**Table 2:** Endogenous mineral content in sweet potato and the estimated amount available for transmetallation or chelation in a thin imaginary cylinder around the wells (scaled down the imaginary computation volume for sweet potato to match the volume used for apple in Table 1).

	Minerals in Common Oxidation States	Gd3 + 157 mg/mL in Scaled down V_well_ = 0.04 mL	Iodine 300 mg/mL in Scaled down V_well_ = 0.04 mL	Na+	K+ ^Ұ^	Mg2+	Ca2+	Mn(2+,3+,4+) ^¶^	Cu2+	Fe(2+,3+) ^¶^
Exogenous or Endogenous Minerals										
Content in **mg/100 g or 220 mL** volume of sweet potato (with density 0.45 g/mL)		**0**	**0**	55	250	20	50	0.3	0.2	0.7
Endogenous content in **μg/mL** of bulk volume in sweet potato		**0**	**0**	240	1100 ^Ұ^	90	215 ^Ұ^	1.3 ^¶^	1	3.5 ^¶^
Exogenous content in **μg** added to carved wells *(per 0.04 mL of well volume)*		6000	12,000	0	0	0	0	0	0	0
Endogenous content in **μg** in a thin imaginary cylinder (v = 0.04 mL) around the wells		**0**	**0**	10	44 ^Ұ^	3.6	8.6 ^Ұ^	0.05 ^¶^	0.04	0.14 ^¶^
K-edges (keV)		50	33	1	3.3	1.3	3.7	**5.9**	**8.0**	**6.4**
Cumulative X-ray absorption cross section (barns/atom) assuming X-ray energies available for photoelectric effects σ < = Abundance × (Z^4^) ^§^		-	-	**σ(expt)** **<<3.5 ×** **10^6^**	**<<** **143 × 10^6^**	**<<** **1.8 ×** **10^6^**	**<<** **34 × 10^6^**	**σ(expt)** <0.05 × 10^6^ = σ (computed)	< 0.7 × 10^6^	< 1.6 × 10^6^

^Ұ^ indicates plausible X-ray absorbers (high abundance or high Z). ^¶^ indicates paramagnetic minerals that alter T_1_ and T_2_* MRI relaxation times of bulk protons. ^§^ denotes inelastic scattering cross sections σ, for absorption at clinical keV range where PE interaction dominates. Note σ(expt) is much less than the σ (computed) depending on how far off the K-edge of a particular metal ion from mean energy of 10–15 keV for X-ray beams with 40–44 kVp is. << stresses that the k-edge is far lower than mean energy of X-ray beam; hence σ(expt) << σ(computed) for alkali and alkaline earth; and < stresses that the k-edge is slightly lower than mean energy of X-ray beam; hence σ(expt) < σ(computed) for transition metals.

## Data Availability

Not applicable.
